# Inorganic Halide Perovskite Quantum Dots: A Versatile Nanomaterial Platform for Electronic Applications

**DOI:** 10.1007/s40820-022-00983-6

**Published:** 2022-12-29

**Authors:** Chien-Yu Huang, Hanchen Li, Ye Wu, Chun-Ho Lin, Xinwei Guan, Long Hu, Jiyun Kim, Xiaoming Zhu, Haibo Zeng, Tom Wu

**Affiliations:** 1 School of Materials Science and Engineering, University of New South Wales, Sydney, 2052 Australia; 2 MIIT Key Laboratory of Advanced Display Materials and Devices, Institute of Optoelectronics and Nanomaterials, College of Materials Science and Engineering, Nanjing University of Science and Technology, Nanjing, 210094 People’s Republic of China

**Keywords:** Inorganic perovskite, Quantum dots, Electronics, Nanocrystals, Quantum confinement effects

## Abstract

Metal halide perovskites have generated significant attention in recent years because of their extraordinary physical properties and photovoltaic performance. Among these, inorganic perovskite quantum dots (QDs) stand out for their prominent merits, such as quantum confinement effects, high photoluminescence quantum yield, and defect-tolerant structures. Additionally, ligand engineering and an all-inorganic composition lead to a robust platform for ambient-stable QD devices. This review presents the state-of-the-art research progress on inorganic perovskite QDs, emphasizing their electronic applications. In detail, the physical properties of inorganic perovskite QDs will be introduced first, followed by a discussion of synthesis methods and growth control. Afterwards, the emerging applications of inorganic perovskite QDs in electronics, including transistors and memories, will be presented. Finally, this review will provide an outlook on potential strategies for advancing inorganic perovskite QD technologies.

## Highlights

- Research progress on inorganic perovskites quantum dots is reviewed from three aspects: physical properties, synthesis approaches, and electronic applications.
- Inorganic perovskite quantum dots have been exploited as either the active layers or the additives in high-performance transistors and memories.
- Challenges and outlook on future advancement of perovskites quantum dots-based electronics are elaborated.

## Graphical abstract

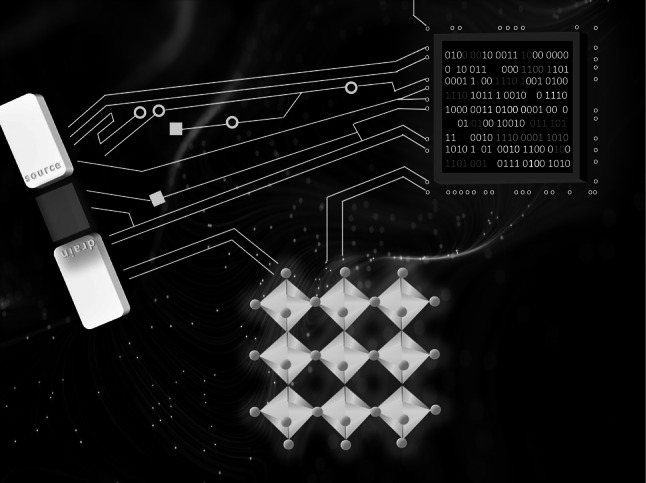

## Introduction

Metal halide perovskites were discovered to exhibit photoconductivity in 1957 [1]. Still, it was not until the last decade that they started to attract enormous attention in the materials science community because of their extraordinary power conversion capability in photovoltaic devices [2–5]. After intensive developments, they have emerged as up-and-coming photovoltaic materials with the highest energy conversion efficiency among thin film materials and promoted the development of high-performance optoelectronic devices [6–9]. In addition, metal halide perovskites have also been exploited as active materials in other high-performance applications such as light-emitting diodes (LEDs), field-effect transistors (FET), and photoelectrochemical catalysis [10–14].

Quantum dots (QDs) with tens of nanometers or smaller dimensions, which feature quantum confinement effects, have been regarded as optical materials distinct from their bulk counterparts and garnered rising research interests [15–17]. Typically, they exhibit eminent physical properties, including size-tunable emission wavelength [17], high photoluminescence quantum yield (PLQY) [18], delta-function-like density of states [19], large optical oscillator strength [20], and low-threshold operation [21]. By virtue of the advantageous characteristics, they are envisioned to find applications in LEDs [22, 23], solar cells [24, 25], lasers [19, 26], medical imaging [27, 28], single-photon source [29, 30] and quantum computing [31, 32]. With their color-tunable emission wavelength and high color purity privilege, major display manufacturers such as Sony, Samsung, and LG have used QDs in commercial products and produced QLED displays [33].

Semiconductor QDs such as CdSe and InP have been widely investigated in the past three decades [34, 35]. Optical absorption and photoluminescence (PL) spectroscopic studies on QDs revealed unique properties such as narrow peak widths of excitons along with exotic properties of biexcitons and higher-order excitons [36]. Although ligands can guarantee phase stabilization of QDs, the excess capping ligands on the QD surface, a typical side effect of colloidal synthesis, may hinder charge transport [37, 38]. As a result, the current of QD solar cells is restricted, and the device performance is often inferior to thin-film alternatives. Therefore, the intricate ligand engineering on QD surfaces is crucial to maintaining both phase stability and carrier transport.

Metal halide perovskite QDs with the hybrid organic–inorganic structure of CH_3_NH_3_PbBr_3_ were firstly reported by Schmidt et al. [39]. However, because of their lower melting point and thermal instability [40, 41], researchers tried to exclude all organic species sensitive to the ambient condition in organic–inorganic hybrid halide perovskites [42]. Kovalenko's group pioneered the research on all-inorganic perovskite QDs of CsPbX_3_ (X = Cl, Br, I or their mixtures) via the hot-injection synthesis method [16], which soon became the focus of the QD community. The size of QDs was controlled by altering reaction temperatures, and the resultant quantum size effect was systematically investigated. This work built the premise for the shape-controlled synthesis of inorganic perovskite QDs. Over the years, the hot-injection synthesis has been optimized with various ligands and precursors to attain better stability and shape control. However, this high-temperature and inert gas-required method is cost-ineffective and limits mass production [43]. Alternatively, researchers developed other methods which are able to synthesize inorganic perovskite QDs under atmospheric conditions, such as ball milling [44], microwave irradiation [45], tip sonication [46], and solvothermal methods [47].

Compared to many traditional semiconductor QDs, inorganic perovskite QDs preserve high-performance features in the presence of high-concentration defects [13, 48]. Furthermore, unlike their bulk and thin film counterparts, the quantum confinement effect leads to strong PL emissions with a high PLQY [49, 50]. They offer other photophysical properties, such as tunable PL emission across the entire spectral range, narrow full width at half maximum (FWHM), large multiple-photon absorption cross section, and low threshold of population inversion. The promising properties enable inorganic perovskite QDs to be used in wide-range applications, including LED, solar cells, photodetectors, nonlinear emission sources, and electro-optic modulators [51–54]. Additionally, they were also exploited in fast X-ray scintillators for ionizing radiation detection [34]. Very recently, the usage of inorganic perovskite QDs has been extended to the area of electronics. Although plenty of work has been done in this emerging area of QD applications, to the best of our knowledge, the progress on inorganic perovskite QD electronics has not been reported in any review.

Despite the benefits listed above, the main challenge confronting inorganic perovskite QDs is long-term structural stability [55]. There are three significant aspects regarding the instability of inorganic perovskite QDs [56]: (i) inorganic perovskite QDs can be degraded by polar solvents or ionic compounds, threatening the long-term structural integrity of QDs [57]; (ii) the ligand-binding is highly ionic, which causes fast ligand desorption and weakens the colloidal state and structural integrity [58]; (iii) light or electric field-induced halide migration causes bond breaking in inorganic perovskite QDs and deteriorates optoelectronic performance [59]. Hence, the maintenance of their structural integrity remains a critical issue.

In this review, we present the state-of-the-art research progress on inorganic perovskite QDs focusing on their electronic applications. The structural and physical characteristics of inorganic perovskite QDs will be thoroughly discussed. Then, the general synthetic methodologies, as well as the size and shape management of inorganic perovskite QDs, will be highlighted. Finally, inorganic perovskite QDs as an active layer in the application of transistors and memory devices will be discussed, which will be followed by a perspective on the future developments of perovskite QD electronics.

## Basic Physical Properties of Inorganic Perovskite QDs

### Lattice Structure of Inorganic Perovskite QDs

**Fig. 1:**
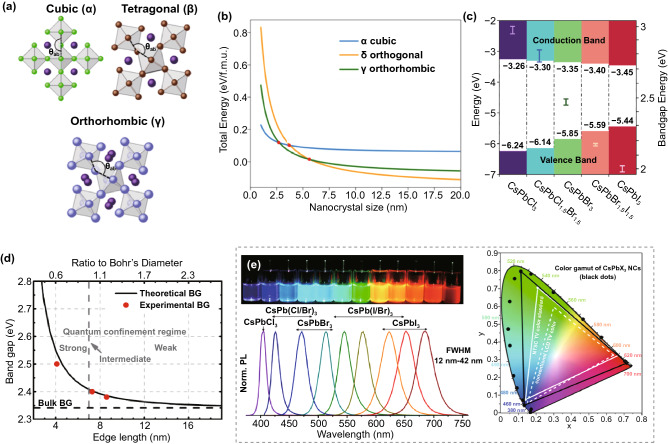
**a** Polymorphic structures of CsPbX_3_. Adapted with permission from Ref. [60]. Copyright 2015, American Chemical Society. **b** Formation energy of α, γ, and δ-phases of CsPbI_3_ as a function of size. Adapted with permission from Ref. [67]. Copyright 2020, American Chemical Society. **c** Energy diagrams of representative inorganic perovskite QDs. **d** Experimental versus theoretical size-dependent bandgap energy of CsPbBr_3_ QDs. Adapted with permission from Ref. [69]. Copyright 2017, American Chemical Society. **e** Photos of inorganic perovskite CsPbX_3_ (X = Cl, Br, I) QD colloidal dispersions, along with PL emission spectra and corresponding CIE chromaticity coordinates. Adapted with permission from Ref. [16]. Copyright 2017, American Chemical Society

Inorganic perovskite QDs crystallize in the ABX_3_ lattice structure (Fig. 1a), where the A-site is cesium ion (Cs^+^), B-site is lead ion (Pb^2+^), and X-site is halide ions (Cl^−^, Br^−^, I^−^ or their mixture) [60]. The structural stability of perovskites is largely determined by the Goldschmidt tolerance factor (TF), which can be calculated as ${\text{TF}} = \frac{{(r_{A} + r_{X} )}}{{\sqrt 2 (r_{{\text{Pb}}} + r_{X} )}}$, where ${r}_{A}$, ${r}_{Pb}$, and ${r}_{X}$ are the ionic radii [48]. In the case of all inorganic perovskites, Cs is too small for the A-site, resulting in thermodynamic instability. TF will be 1 in an ideal close-packing case. TF values of CsPbBr_3_ and CsPbI_3_ are 0.9 and 0.89, respectively, indicating their structural instability. Aside from the common ABX_3_ structure, there is another perovskite structure with the general formula A_2_BX_6_ which removes half of the B sites and is known as double perovskites [61]. The B-site cation is coordinated with six X-site anions to form a corner-sharing [BX_6_] octahedral configuration, with the A-site cation further occupying the cuboctahedra cavity.

It is well known that inorganic perovskites have three main phases: cubic, tetragonal, and orthorhombic [62–65]. The preferentially stable phase at room temperature is dramatically related to the size of inorganic perovskites. For example, the tetragonal phase is most stable for bulk CsPbI_3_ at room temperature but will convert to a non-perovskite orthorhombic phase due to low formation energy [66–68]. However, when reducing CsPbI_3_ to the QD scale, the surface energy and the total Gibbs free energy for the non-perovskite orthorhombic phase become more considerable, thereby inhibiting the detrimental phase transition (Fig. 1b). Hence, the cubic and tetragonal phases of CsPbI_3_ QDs are more thermodynamically favorable than the solar-inactive non-perovskite orthorhombic phase [67]. Furthermore, the phase transformation can be triggered via various external conditions. CsPbI_3_ QDs with highly metastable cubic phase readily convert to the stable non-perovskite orthorhombic phase via adding extra polar additives capable of removing the surface ligands, leading to the loss of strong-emitting properties. CsPbBr_3_ and CsPbCl_3_ QDs undergo phase transformation at elevated temperatures.

### Quantum Confinement Effect

One of the most essential characteristics of QDs is quantum confinement, which leads to the quantization of energy due to their nanoscale sizes. This phenomenon occurs when the wavefunctions of electrons and holes are shrunk down to dimensions smaller than the excitonic Bohr radius [70]. The confinement energy can be estimated as $\Delta E=\frac{{\hslash }^{2}{\pi }^{2}}{2{m}^{*}{r}^{2}}$, where $\hslash$ is Planck constant, m* is the exciton reduced mass which relates to the Bohr radius through the effective mass approximation, and r is the particle radius. The excitonic Bohr radii of inorganic halide perovskites are quite small, in the range of a few nanometers [16]. By controlling the size of inorganic perovskite QDs, their bandgap can be tunable, further influencing their PL emission wavelengths. According to the study by Butkus et al., when the particle size decreases from 8.5 to 4.1 nm, the bandgap of CsPbBr_3_ QDs increases from 2.37 to 2.5 eV (Fig. 1d), accompanied by obvious blue shifts in the PL spectra [69]. However, once the size of inorganic perovskite QDs surpasses the Bohr radius, the quantum confinement effect is no longer remarkable [71].

The size and halogen of CsPbX_3_ QDs can affect carrier transport, spin relaxation, and phonon behaviors. Li et al. studied the spin relaxation of CsPbI_3_ and CsPbBr_3_ QDs with different sizes [72]. Both kinds of QDs feature decreased spin lifetime along with reduced dimensions. However, compared with their bulk control samples, the lifetime of CsPbI_3_ and CsPbBr_3_ QDs is prolonged and shortened, respectively, indicating the different spin-flip mechanisms between bulks and QDs. The Elliott–Yafet (E–Y) phonon scattering mechanism is prominent in bulk CsPbI_3_ but absent in bulk CsPbBr_3_. In the form of QD, the suppression of E–Y scattering consequently leads to a long spin lifetime for CsPbI_3_ QDs. On the other hand, the quantum size-induced spin relaxation mechanisms, including surface scattering, electron–hole exchange, and spin–spin interaction with surface dangling bond spins, will shorten the spin lifetime of CsPbBr_3_ QDs. This size scaling mechanism demonstrated the influence of material dimensionality and carrier diffusion on luminescence efficiency and carrier recombination kinetics [73].

### Optical and Electronic Properties

Inorganic perovskite QDs exhibit strong and narrow PL light emissions [74, 75]. Compared with traditional semiconductor QDs such as CdSe, CdS, or PbS, inorganic perovskite QDs can achieve more than 99% PLQY without any surface passivation by the wide-bandgap epitaxial shells [76–78]. This remarkable trait is a symptom of the high defect tolerance in inorganic perovskite QDs, which derives from their electronic structure and the bandgap between two antibonding orbitals [71].

Inorganic perovskite QDs also exhibit wavelength-tunable emission across the entire visible range, which can be mainly controlled by the halide composition. Protesescu et al. reported that PL emission spectra of CsPbX_3_ QDs could be shifted from 410 to 685 nm by substituting or mixing different halide anions (X = Cl, Br, or I), as shown in Fig. 1e [16]. The facile optical property is related to the electronic structure of inorganic perovskite QDs. Notably, the upper valence band (VB) of inorganic perovskite QDs comes primarily from the *np*^6^ orbitals of X-site anion (*n* = 3, 4, 5 for Cl, Br, I, respectively) and *ns*^2^ orbitals of B-site cation (*n* = 6 for Pb). The lower conduction band (CB) results from dominant contributions from p orbitals of B-site cation and *np*^6^ orbitals of X-site anion [79]. Consequently, when the halide changes from I (5*p*^6^) to Br (4*p*^6^) to Cl (3*p*^6^), the energy of the X-site *np*^6^ orbitals falls and the valence band maximum increases (Fig. 1c), leading to the tunability of the bandgap of inorganic perovskite QDs. In addition to X-site replacement, A- or B-site modification of halide perovskite QDs can modify the bandgap. For instance, if the molecule occupying the A-site changes from formamidinium (FA^+^), methylammonium (MA^+^) to Cs^+^, the tilting angle of Pb-X-Pb bonds tends to decrease and consequently causes blue shifts in the bandgap [80]. If Pb^2+^ at B-site is replaced with Sn^2+^, the enhanced electronegativity will decrease the perovskite bandgap from 2.33 to 2.15 eV for bromine perovskites and from 1.55 to 1.3 eV for iodide ones [81, 82].

Carrier mobility, diffusion length, and carrier lifetime are three key parameters determining the electronic properties of optoelectronic devices. For perovskite QDs, although different experimental techniques often render different values, their composition-dependent carrier mobilities are generally in the same trend as polycrystalline thin films. Take all-inorganic perovskites CsPbX_3_ as examples, both the theoretical and experimental results verified that CsPbI_3_ QDs have the highest carrier mobility of 20 cm^2^ V^−1^ s^−1^ [83], followed by CsPbBr_3_ (~ 2.1 cm^2^ V^−1^ s^−1^) [84] and CsPbCl_3_ [85–87]. Likewise, as suggested by TRPL results, CsPbI_3_ QDs also own the longest lifetime of 29 ns, while CsPbCl_3_ has the shortest lifetime of 1 ns [16]. Diffusion length is proportional to both carrier mobility and lifetime, which can be expressed by ${L}_{D}=\sqrt{{D}_{\tau }}$, where $D$ is the diffusion coefficient and *τ* is the lifetime of the excited carrier [88]. In detail, diffusion coefficient is defined as $D=\frac{\mu {k}_{\text{B}}T}{q}$, where $\mu$ is the charge carrier mobility, ${k}_{B}$ is the Boltzmann constant, $T$ is the temperature, and $q$ is the charge of an electron. Hence, according to carrier mobility and lifetime results, CsPbI_3_ also has the longest diffusion length.

Inorganic perovskite QDs are ideal host matrices for enabling quantum cutting with rare-earth ions. Song et al. demonstrated in 2017 that doping with rare-earth ions into CsPbX_3_ QDs led to a PLQY value of nearly 150% [89]. Moreover, the rare-earth-doped highly luminescent lead-free double perovskite QDs have been reported. Liu et al. first unveiled the doping effects of the rare earth element, Tb^3+^, on Cs_2_AgInCl_6_:Bi QDs, and the results were proved by both experimental structural analysis and first-principle calculation [90]. In their study, altering the doping quantity of Tb^3+^ changed the emission colors of Bi-doped Cs_2_Ag(In_1−*x*_Tb_*x*_)Cl_6_ from green to orange. Wang et al. presented another co-doping strategy [91]. After co-doping Bi^3+^ and Ce^3+^ into Cs_2_Ag_0.4_Na_0.6_InCl_6_, the internal quantum efficiency increased from 89.9 to 98.6%. Additionally, Gamelin's group reported that the highest PLQY based on Yb^3+^-doped CsPb (Cl_1−*x*_Br_*x*_)_3_ perovskite QDs reached ~ 200% [92], which comes from the quantum cutting effect of Yb^3+^.

## Synthesis Methods

Synthesis methods of inorganic perovskite QDs are mainly based on solution reaction and can be divided into direct synthesis and post-synthesis [93, 94]. Direct synthesis comprises ligand-assisted reprecipitation (LARP), emulsion synthesis, hot injection, ultrasonication, microwave, solvothermal, and chemical vapor deposition (CVD). These approaches produce QDs of various forms, including nanospheres and nanocubes. Post-synthesis is an alternative approach and uses QDs that have already been manufactured as templates. Ion exchange and phase transformation are prominent examples of post-synthesis techniques.

### Direct Synthesis Methods

In general, direct synthesis methods can be divided into three categories, solution based, solid based, and gas based. The solution-based synthesis includes LARP, emulsion synthesis, hot injection, ultrasonication, microwave, and solvothermal. In the typical solvent-free solid-based method, mortar and pestle were used to ground precursors in a mechanochemical process. The gas-based method mainly relies on chemical vapor deposition (CVD).

**Fig. 2:**
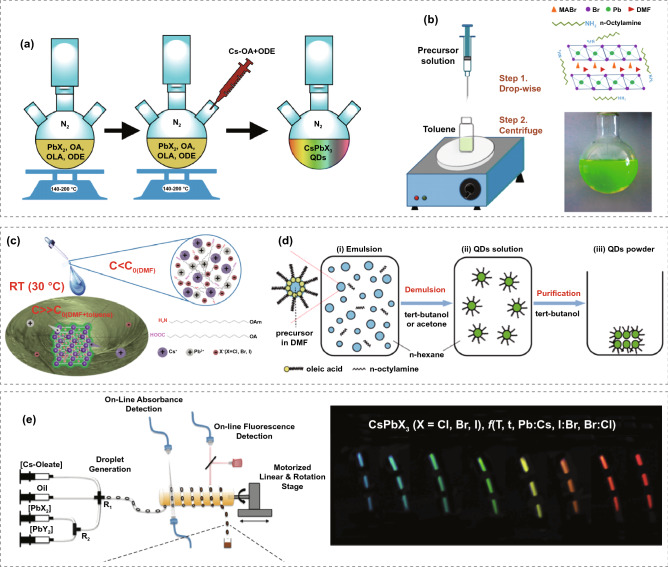
**a** Schematic diagram of the hot-injection method for synthesizing inorganic perovskite QDs. **b** Schematic diagram of LARP method for inorganic perovskite QDs fabrication. Adapted with permission from Ref. [95]. Copyright 2015, American Chemical Society **c** Schematic diagram of the ligand-assisted method. Adapted with permission from Ref. [96]. Copyright 2016, Wiley–VCH. **d** Schematic diagram of the mechanism of the emulsion method. Adapted with permission from Ref. [97]. Copyright 2015, American Chemical Society. **e** Schematic diagram of the microfluidic method and the photo of obtained CsPbX_3_ QDs under UV excitation. Adapted with permission from Ref. [104]. Copyright 2016, American Chemistry Society

Protesescu et al. synthesized inorganic perovskite QDs for the first time in 2015 by adopting the hot-injection method [16]. Figure 2a describes the scheme of hot-injection synthesis, in which a certain amount of Cs-oleate precursor was injected into a three-neck flask containing lead halide (PbX_2_) salts dissolved in 1-octadecene (ODE), oleic acid (OA), and oleylamine (OAm) at 140–200 °C under the protection of inert gas. After a few seconds, CsPbX_3_ forms through rapid cool down in an ice bath. In this process, OAm and OA act as surfactants and provide the function to solubilize PbX_2_ salts during the synthesis and stabilize CsPbX_3_ QDs at the final stage. The ratios among different chemicals and reaction temperature can determine the size and shape of QDs, which can further influence their optical properties.

The original concept of LARP was proposed by Schmide et al. [39]. They demonstrated the preparation of MAPbX_3_ perovskite QDs via a facile colloidal strategy using ligands with a medium-sized chain to stabilize the colloidal phase. Inspired by this, Zhang et al. further modified the process to prepare MAPbX_3_ QDs and named it LARP [95]. The mechanism of LARP is shown in Fig. 2b. In a typical process, the precursor, which is a mixture of MABr, PbBr_2_, OA, and *n*-octylamine (OM) dissolved in dimethylformamide (DMF), is poured into a vessel containing toluene while stirring, and supersaturate precipitation is induced at room temperature to form a yellow-green QDs dispersion, thereby overcoming the temperature limitations of hot-injection methods. Li et al. also developed a similar method leveraging ligand assistance to generate inorganic perovskite QDs under the room temperature (Fig. 2c) [96]. The obtained inorganic perovskite QDs have a monoclinic phase structure, unlike the conventional product of hot injection. The emulsion synthesis method includes the emulsion formation and demulsion processes for producing inorganic perovskite QDs, as illustrated in Fig. 2d [97]. The surfactants, polar, and nonpolar solvents are mixed to start the emulsion, followed by adding the demulsifier into the immiscible solution to crystallize QDs. Taking Yang et al.'s study as an example, they first dissolved CsBr in deionized water (diH_2_O) and PbBr_2_ in DMF. Then, they made the "oil phase" by mixing oleic acid with *n*-octylamine in 10 mL hexane. After that, a dropwise mixture of the CsBr-diH_2_O and PbBr_2_-DMF solution was added into the oil phase. This caused the oil phase to progressively transform from clear to a faint white color, which resulted in the formation of an emulsion. Finally, acetone was used to initiate a demulsion process, and the QDs were collected after centrifuging the mixture [98]. Although LARP and emulsion techniques have similar mechanism, their supersaturated environments are distinct. Specifically, the solvent mixing in LARP would promote a change in solubility and result in the nucleation of QDs. In contrast, in the emulsion method, the crystallization of QDs would be triggered by microreactors that result from solvent mixing.

The ultrasonication method to produce highly luminescent QDs was firstly applied by Polavarapu's group [46]. By placing the mixture including Cs_2_CO_3_, PbX_2_, OA, and OAm in ODE under the tip sonication, the uniform inorganic perovskite QDs with PLQY ranging from 10 to 92% were formed rapidly with the assistance of sufficient heat provided by ultrasonication. Further studies have also been done to increase both PLQY and long-term stability of inorganic perovskite QDs generated via the ultrasonication method [46, 99, 100]. Furthermore, Polavarapu's group revealed a series of self-assembly and shape control of inorganic perovskite QDs via using the ultrasonication synthetic approach. By prolonging ultrasonication time, the initially formed inorganic perovskite QDs are assembled into nanowires via oriented attachment [101]. Moreover, the inorganic perovskite nanowires generated via the ultrasonication method can undergo halide exchange and shape fragmentation into low aspect ratio nanorods with the addition of PbX_2_ precursor solution prepared by mixing PbX_2_ salts, OA, and OAm in hexane [102].

The solvothermal method and microwave-assisted method to fabricate QDs were firstly reported by Zhang’s group [45, 47]. The solvothermal synthesis method is featured with its simplicity as all the precursors are mixed and heated. The QDs synthesized by the solvothermal method under a high-pressure environment exhibited high-quality crystallinity. Afterwards, they further proposed another microwave-assisted QDs synthesis approach that is efficient and quick. By applying the benefits offered by microwave heating, which include a high heating rate and low overall energy consumption [45, 103], CsPbX_3_ nanocrystals with cubic morphology in a uniform size can be achieved. In addition, various controllable morphologies of CsPbX_3_ nanocrystals, such as nanoplate and nanorod, may be fabricated by adjusting reaction parameters such as reaction temperature, heating rate, and pre-dissolution precursors. Compared with the hot-injection method, in which precursors need to be prepared separately in an inert atmosphere, the solvothermal and microwave-assisted approaches are much simpler since all precursors are combined in the air without any further pretreatment.

The microfluidic method for forming inorganic perovskite QDs shown in Fig. 2e was designed by Lignos et al. [104]. Different chemical reagents were provided via individual syringes and transferred to a cross-mixer for synthesizing inorganic perovskite QDs under the desired temperature. Specifically, various parameters, including precursor types, solution concentrations, flow rate, temperature, and reaction time, can be precisely controlled, providing enormous possibilities for scale-up manufacturing. Besides, Li et al. applied the microfluidic method to obtain novel long-armed hexapod structures of CsPbBr_3_ [105]. The CsPbBr_3_ hexapods were synthesized by a segmented-flow microfluidic reactor initially. After mixing OAm and OA surface ligands with ODE at 180 °C, the carrier oil should be separated, and the solution was stirred for another 36 h at room temperature. Then, the hexapod structure will form by oriented attachment. The multipod nanostructures for perovskite have been reported earlier, but the arm–core ratio (0.2) and arm length (10 nm) were much lower than Li's work (arm length up to 360 nm and arm–core ratios up to 6.0) [106, 107].

Comparisons may be made among various synthesis methodologies. The hot-injection method is the most reliable and popular one among all methods mentioned above. Owing to the high-temperature growth process, the resulting QDs usually possess a uniform size and shape with high crystallinity. In contrast, the LARP and emulsion synthesized products feature relatively low crystallinity and stability because of low reaction temperature and polar solvent, which severely degrades or forms defective QDs [58]. However, it still has some merits like room-temperature fabrication and short reaction time. The ultrasonication, microwave-assisted, and solvothermal reactions are advantageous for the excellent control of the QD size by reaction time. Still, a large amount of QDs is wasted during the separation and purification processes. The microfluidic method is the latest one modified from the hot-injection method. The process can be precisely controlled to obtain QDs with desirable absorption and emission spectra, which possess great potential to be commercialized [93].

Apart from the solution-based synthesis method, solid-based and gas-based ones have also been created to fabricate inorganic perovskite QDs. The solid-phase synthesis was first reported by Jana et al. [108]. It is a solvent-free and mechanochemical process in which the mortar and pestle are used to ground CsBr, PbBr_2,_ and n-octylammonium bromide at room temperature. Although the synthetic procedure is straightforward, the PLQY of obtained CsPbBr_3_ QDs is only 13%, which is far lower than that of typical solution-based synthesis. Huang et al. developed another interesting solid-based strategy, in which CsPbBr_3_ QDs were formed inside the transparent glass with Cs, Pb, and Br elements through femtosecond laser-induced in situ crystallization [109]. Meanwhile, the CsPbBr_3_ QDs inside glass can be fabricated and reversibly modified by femtosecond laser irradiation and thermal annealing. Therefore, this strategy is compatible with 3D laser printing to grow highly luminescent inorganic perovskite QDs arrays and patterns by computer-controlled 3D translation laser stage. In addition, the gas-based method mainly relies on chemical vapor deposition (CVD) [110, 111]. The inorganic perovskite QDs can grow within the glass tube holding the precursors of CsX and PbX_2_ in the presence of argon flow. The CVD-produced inorganic perovskite QDs have excellent optical properties and are widely employed in photodetectors. [112].

### Post-Synthesis Modification of NP Composition and Morphology

Post-synthesis entails the use of chemical procedures to modify pre-made inorganic perovskite QDs, hence providing unique properties that are not feasible with direct synthesis techniques. This part will address two post-synthesis techniques: ion exchange and phase transformation.

**Fig. 3:**
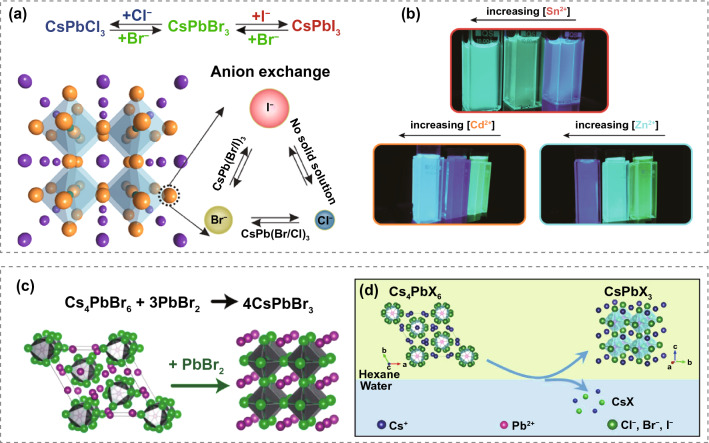
**a** Schematic diagram of halide exchange in inorganic perovskite QDs. Adapted with permission from Ref. [122]. Copyright 2015, American Chemistry Society. **b** Photos of CsPbBr_3_ QD colloidal dispersion after reacting with divalent cation bromide salts under the UV excitation. Adapted with permission from Ref. [116]. Copyright 2017, American Chemistry Society. **c** Schematic diagram of transformation from Cs_4_PbX_6_ to CsPbX_3_ QDs. Adapted with permission from Ref. [123]. Copyright 2017, American Chemistry Society. **d** Schematic diagram of chemical conversion from Cs_4_PbX_6_ to CsPbX_3_ QDs. Adapted with permission from Ref. [124]. Copyright 2017, American Chemistry Society

The ion exchange can be divided into anion and cation exchanges. Generally, the anions (X^−^) in CsPbX_3_ can be easily replaced with other halide ions because of the ionic structure of inorganic perovskite QDs [94, 113, 114]. For instance, Nedelcu et al. applied various halide precursors for as-synthesized inorganic perovskite QDs to deliver anion exchange, tuning the PL spectra across the entire visible range without distinct morphology changes (Fig. 3a). Additionally, the cation (Pb^2+^) can also be exchanged with other divalent cations, such as Mn^2+^, Zn^2+^, and Cd^2+^, to modify the properties of QDs. Many groups presented different divalent cation exchanges in their inorganic perovskite QD works. Son et al. reported the photoinduced post-synthesis method of Mn doping in inorganic perovskite QDs. In the presence of a tiny quantity of dissolved manganese acetate in dihalomethane, Mn^2+^ was successfully doped in CsPbX_3_ QDs under exposure to UV light [115]. Stam et al. further demonstrated a simple way of cation exchange [116]. They combined as-obtained CsPbBr_3_ QDs with metal bromide (SnBr_2_, ZnBr_2_, and CdBr_2_) and oleylamine in toluene. After stirring overnight, partial Pb^2+^ can be replaced by foreign metal ions with smaller ionic radius, contributing to lattice contraction of inorganic perovskite QDs and reflecting blue shifts in PL spectra **(**Fig. 3b**)**. In addition to maintaining the inorganic perovskite QDs as the structure of CsPbX_3_, structure transformation can also undergo during the post-synthesis treatment. Both Akkerman et al. and Wu et al. demonstrated the transformation from Cs_4_PbX_6_ nanocrystals to monodispersed CsPbX_3_ QDs. Specifically, Akkerman et al. added extra PbX_2_ precursor into the colloidal dispersion of as-prepared Cs_4_PbX_6_ nanocrystals, while Wu et al. tried to strip CsX from Cs_4_PbX_6_ nanocrystals with the assistance of high solubility of CsX in water. Palazon et al. also realized such structure transformation via the process of thermal annealing or Prussian blue post-treatment [117]. Interestingly, CsPbX_3_ QDs can also be changed to Cs_4_PbX_6_ nanocrystals by adding organic thiol compounds, as reported by Liu et al. [118, 119]. Due to the strong affinity between thiol and PbX_2_, PbX_2_ can be swept away from CsPbX_3_ QDs, inducing the structure conversion. Furthermore, Udayabhaskararao et al. successfully achieved the reversible transformation between CsPbX_3_ QDs and Cs_4_PbX_6_ nanocrystals by controlling the OA/OAm ratio [120]. Yang et al. studied both the structure and morphology change of pristine Cs_4_PbBr_6_ nanocrystals through the post-treatment process [121]. They introduced a certain amount of water into the Cs_4_PbBr_6_ nanocrystals dispersed in a nonpolar solvent. Then monodisperse CsPbBr_3_ nanorods can be prepared by controlling the concentration of Cs_4_PbBr_6_ nanocrystals and reaction time.

### Size and Shape Control

Via judiciously choosing synthetic methods or reaction parameters, nanoscale inorganic perovskites can be produced with different shapes, such as nanowires, nanocubes, nanosphere, and nanoplates [94]. Among them, the inorganic perovskite QDs with cubic morphology are the most common products [16, 45, 125].

**Fig. 4:**
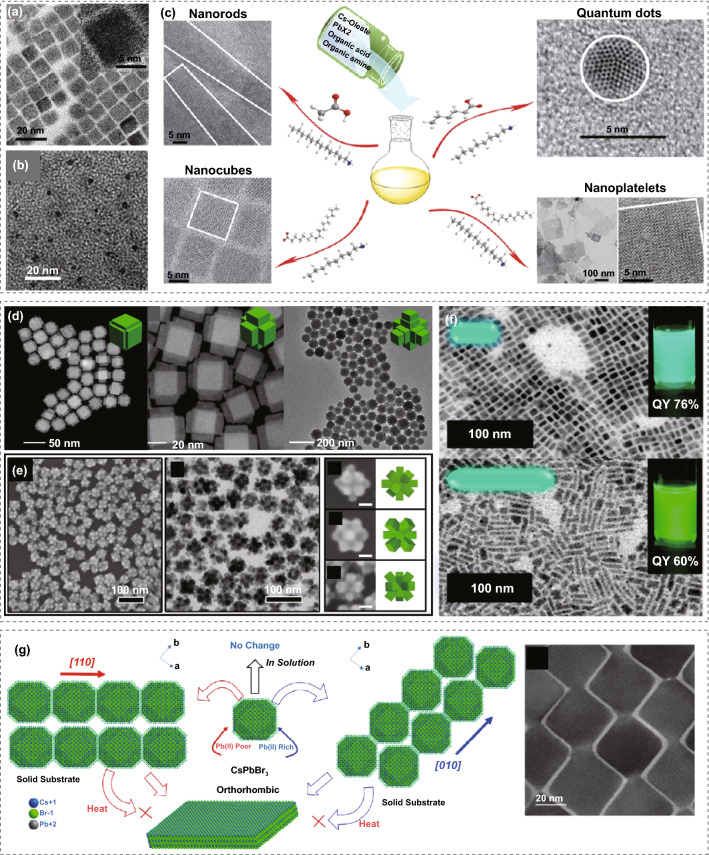
**a** Transmission electron microscopy (TEM) image of cuboid CsPbBr_3_ QDs. Adapted with permission from Ref. [96]. Copyright 2016, Wiley–VCH. **b** TEM images of CsPbBr_3_ nanospheres. Adapted with permission from Ref. [126]. Copyright 2016, American Chemistry Society. **c** Schematic diagram of different morphologies of inorganic perovskite nanocrystals produced by varying the ligands. Adapted with permission from Ref. [127]. Copyright 2016, American Chemistry Society **d** TEM of CsPbBr_3_ and CsPbI_3_ nanocrystals formed under different reaction temperatures. Adapted with permission from Ref. [107]. Copyright 2019, American Chemistry Society. **e** Scanning electron microscope (SEM) and TEM image of CsPbBr_3_ dodecapods nanoflowers. Adapted with permission from Ref. [106]. Copyright 2018, Elsevier Publishing. **f** SEM image of CsPbBr_3_ nanorods formed by tip sonification. Adapted with permission from Ref. [128]. Copyright 2019, American Chemistry Society. **g** Schematic diagram of directional connection of CsPbBr_3_ QDs. Adapted with permission from Ref. [129]. Copyright 2020, American Chemistry Society

Turning the reaction temperatures, ligand concentrations, and ligand types are the most used approaches to produce CsPbX_3_ nanomaterials with different shapes. Liang et al. first successfully obtained CsPbBr_3_ nanospheres (Fig. 4c) by modifying the quantity of OA and OAm to 0.6 and 0.3 mL, respectively, during the hot-injection reaction process [126]. Moreover, Sun et al. applied alkyl ligands with various chain lengths to easily tailor the shape of CsPbX_3_ (Fig. 4c) [127]. In addition, the size control was also revealed by Li et al. [96]. They reported CsPbX_3_ nanocrystals fabricated through ligand-assisted methods at 0 °C with an ultra-small sphere shape and a nearly amorphous structure. Furthermore, during the hot-injection process, Zhang's group presented results that solvents with different polarity would also impact the nucleation and growth of QDs by an oriented attachment mechanism, indicating that low-polarity solvents may lead to better control of growth [130].

CsPbX_3_ nanocrystals with other novel shapes have also been developed. Peng et al. synthesized CsPbX_3_ nanocrystals with armed hexapod structures [107]. The synthesis strategy is based on the hot-injection method with the addition of halide seed clusters as a reaction medium. The reaction temperature can control the length and growth direction of the arms, as shown in Fig. 4d. In another work, Chen et al. produced CsPbBr_3_ nanoflowers with a dodecapod-branched structure. Under the Cs-rich environment, cuboctahedral Cs_4_PbBr_6_ seeds underwent structure transformation and formed CsPbBr_3_ nanoflowers with strong PL [106]. The TEM image of CsPbBr_3_ nanoflowers is shown in Fig. 4e. In addition to the multifacet structure, Li et al. fabricated CsPbBr_3_ nanorods with widths of 5 nm and lengths of 10 or 25 nm [128]. The nanorods were produced by substituting butanol for the commonly used nonpolar solvent ODE. The reaction was carried out under the application of tip ultrasonication, as shown in Fig. 4f. Connected nanocrystals with shape modulation were reported by Hudait et al. [129]. The connection schematic is presented in Fig. 4g. By manipulating the composition of precursors, the connection orientation between QDs may be altered to form various geometries. When excess Pb precursor is applied, corner-wise connected QDs will lead to zigzag-shaped one-dimensional (1D) nanostructures. On the other hand, under the less Pb condition, the side-wise connection of QDs occurs, forming nanorods. The connection and transformation process can be ceased by adding ligands or heating. Moreover, inorganic perovskite QDs can be bonded to other nanocrystals to create heterostructures. Imran et al. presented a new type of perovskite-chalcogenide nanocrystal heterostructures, CsPbBr_3_–Pb_4_S_3_Br_2_, prepared by two-step direct synthesis methods [131]. Via post-synthesis halide exchange, CsPbCl_3_–Pb_4_S_3_Br_2_ and CsPbCl_3_-Pb_4_S_3_Br_2_ nanocrystals were also achieved. In those structures, a single inorganic perovskite nanocrystal shares an epitaxial interface with a lead chalcohalide nanocrystal because of the favorable matching of the corresponding Pb sublattices.

Varying reaction parameters can also determine the size control of inorganic perovskite nanocrystals. Almeida et al. reported that the size of CsPbBr_3_ QDs can be changed from 4.0 to 16.4 nm by increasing both the OAm/OA ratio and reaction temperature [132]. Besides, Dong et al. reported the size variation of CsPbBr_3_ through tuning the Br-to-Pb ratio in the precursor solution or reaction temperature. While maintaining a constant Br-to-Pb ratio in the percussor solution, the size of CsPbBr_3_ QDs decreased with increasing temperature [133]. The size tuning strategies and synthesis parameters of inorganic perovskite QDs in the literature are summarized in Table 1.

**Table 1:** Summary of some representative works on size control of inorganic perovskite QDs

Materials	Synthesis method	Size (nm)	Precursor	Temperature (°C)	Ligand	PLQY (%)	Refs.
CsPbI_3_	Hot-injection	13.6	Cs_2_CO_3_/PbI_2_	170	OA OAM	80	[134]
		12	Cs_2_CO_3_/PbI_2_		OA OAM 2,2’-Iminodibenzoic acid (IDA)	95	
CsPbI_3_	Hot-injection	5.7	Cs_2_CO_3_/PbI_2_	140	OA OAM	N/A	[135]
		7.6		160			
		13.9		180			
CsPbBr_3_	Hot-injection	9.5	Cs/Pb = 0.77 Br/Pb = 2.6 (Cs_2_CO_3_/PbBr_2_)	190	OA OAM	85–95	[133]
		6.2	Cs/Pb = 0.75 Br/Pb = 3.1 (Cs_2_CO_3_/PbBr_2_)			85–95	
		5.3	Cs/Pb = 0.75 Br/Pb = 3.2 (Cs_2_CO_3_/PbBr_2_)			80–90	
		3.7	Cs/Pb = 0.79 Br/Pb = 3.5 (Cs_2_CO_3_/PbBr_2_)			80–90	
		8.8	Br/Pb = 10 (Cs_2_CO_3_/PbBr_2_)	190		N/A	
		5.3		160			
		4.1		140			
		3.7		80			
CsPbBr_3_	Hot-injection	7.2	Cs_2_CO_3_ PbBr_2_	180	OA OAM	N/A	[136]
		9.0		160			
		11		140			
		12		120			
CsPbBr_3_	Microfluidic methods	13	Flow velocity = 15 (mL h^−1^) CsBr PbBr_2_	25	OA OAM	N/A	[137]
		1.4	Flow Velocity = 15 (mL h^−1^) CsBr PbBr_2_	− 15	OA OAM		
		3.5	Flow Velocity = 1 (mL h^−1^) CsBr PbBr_2_	25	OA OAM		
CsPbCl_3_	Microemulsion method	3.5	Cs_2_CO_3_/PbCl_2_	RT	OA OAM	N/A	[138]
CsPbCl_3_	Hot-injection	5	Cs_2_CO_3_/PbCl_2_	90	OA OAM	N/A	[139]
CsPbCl_3_	Hot-injection	9	Cs_2_CO_3_/PbCl_2_	210	OA OAM	2.4	[140]
		11	Cs_2_CO_3_/PbCl_2_ NiCl_2_			96.5	

## Inorganic Perovskite QDs Based Electronics

Recently, the perovskite-based FETs and light-stimulated neuromorphic devices gained lots of scientific attention. Compared with halide perovskite bulk films, halide perovskite QDs possess the advantages of size-dependent bandgap tunability from the quantum confinement effect, ideal unit photoluminescence quantum yield approaching 100%, and free phase segregation in mixed halide components [84, 141]. In addition, organic capping ligands in perovskite QDs offer excellent endurance performance for memristor of 2 × 10^6^ cycles [142]. For transistors, electron current dominates the performance in perovskite QDs films under dark condition rather than potentiostatic polarization in bulk films. This impressive current amplification avoids ion accumulation at interface of transistors, resulting in remarkable hole mobility of ~ 10^−3^ and on–off ration of ~ 10^4^ [143]. The other outstanding properties of QDs, including excellent processability and defect tolerance, promise their future possibility in electronics. For instance, perovskite thin films are deposited through a two-step spin coating with precursor and antisolvent dropping, followed by an annealing process [144, 145], in which complicated crystal nucleation and growth mechanism can be involved during film deposition. Thus, precise deposition method control is crucial to the quality and reproducibility of the film. In contrast, high crystallinity of perovskite QDs can be obtained during synthesis in a more straightforward approach. Subsequently, a layer-by-layer deposition is applied, resulting in high reproducibility [146].

Other grand challenges remain regarding device stability, power consumption, bandwidth limit, and so on. To address the problems, inorganic perovskite QDs with extraordinary optoelectronic properties have been considered promising candidates. Here, we summarize the state-of-the-art works involving inorganic perovskite QDs as the active material in transistors and memory devices, which present potential for next-generation electronic applications.

### Inorganic Perovskite QDs Based and QDs-Containing Transistors

Silicon-based semiconducting materials have served as the dominant active layer for transistors in past decades, but there is still a need for emerging materials with low-cost and large-area manufacturing capabilities [147]. In recent years, inorganic perovskite QDs have been defined as one of the “star” materials owing to their outstanding features such as tunable bandgap, shallow defects, ambipolar charge transport and fast ions migration [148]. Furthermore, inorganic perovskite QDs may be produced using low-cost solution processing, allowing for the development of stretchable and flexible integrated transistors, which is a benefit compared to crystalline Si and group III–V semiconductors [13]. In addition, most research on inorganic perovskite QDs has focused on LEDs, solar cells, and photodetectors, while QD electronics are still in their infancy [149–151]. Hence, there remains lots of room for perovskite QDs to develop into next-generation transistor materials.

FET is a prevalent type of electronic device with three terminals, i.e., source, drain, and gate. Mostly, inorganic perovskite QDs serve as the charge transport channel layer in a FET, which is a voltage-controlled electrical switch [152]. The source and drain electrodes are directly in contact with the perovskite layer, while the gate electrode is separated by a dielectric layer. The current flow from the source to the drain is reversibly controlled by applying a voltage bias to the gate, and the conductivity of the perovskite layer is altered correspondingly.

**Fig. 5:**
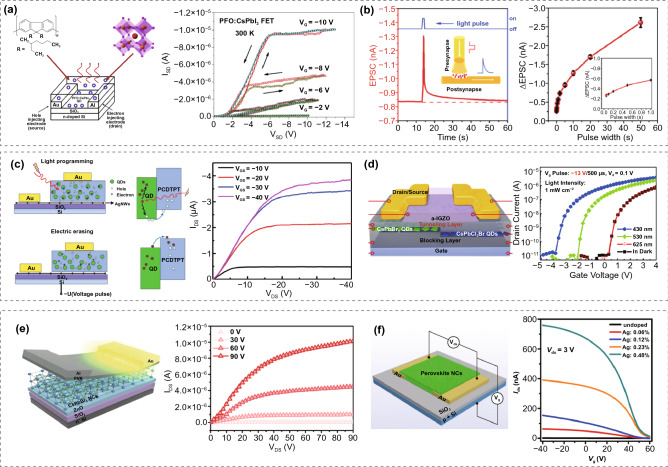
**a** The structure and output curves of the PFO:CsPbI_3_ FET. Adapted with permission from Ref. [153]. Copyright 2017, Elsevier Publishing. **b** Schematic diagram demonstrating the mechanism of CsPbBr_3_ QD-based light simulated synaptic FET and the measurement results. Adapted with permission from Ref. [157]. Copyright 2019, Wiley–VCH. **c** Operating mechanism of the photonic memory device under light programming and electric erasing processes, along with its output characteristic curves. Adapted with permission from Ref. [158]. Copyright 2020, Royal Society of Chemistry **d** Schematic diagram of the stepped inorganic perovskite QDs-based floating-gate transistor structure and its transfer curves under light illumination with different wavelengths. Adapted with permission from Ref. [162]. Copyright 2022, American Chemistry Society. **e** Schematic diagram of the CsPbBr_3_ QD-based light-emitting field-effect transistor and its transfer curves. Adapted with permission from Ref. [163]. Copyright 2020, American Chemistry Society. **f** Schematic diagram of Ag^+^-doped CsPbBr_3_ QDs FET device structure and its output characteristics. Adapted with permission from Ref. [164]. Copyright 2019, American Chemistry Society

At the initial stage, the inorganic perovskite QDs are mostly blended with organic semiconductors to increase the charge separation efficiency to achieve high photoresponsivity. In 2017, Aleshin et al. first blended CsPbI_3_ nanocrystals with a conjugated polymer poly[9,9-bis-(2-ethylhexyl)-9H- fluorene-2,7-diyl] (PFO) [153]. The FET mobility was 0.19 cm^2^ V^−1^ s^−1^ at 300 K in the linear regime of transfer characteristics (Fig. 5a). In another work published in the same year, Chen et al. fabricated top contact/bottom gate (TC/BG) FET based on a thin film combining CsPbBr_3_ QDs and dinaphtho-[2,3-b:2′,3′ -f] thieno[3,2-b] thiophen (DNTT), and a high photoresponsivity of 1.7 × 10^4^ A W^−1^ and an ON/OFF ratio of 8.1 × 10^4^ were recorded [154]. Such inorganic perovskite QD-organic semiconductor hybrid phototransistors exhibit outstanding stability after 100 days of storage in ambient air [154]. However, due to the nanoscale crystal size and insulating organic ligands capping on the surface [155], pure CsPbX_3_ QDs films usually feature low conductivity, thereby restricting their application in electronic devices.

Recently, artificial synaptic devices combining multiple excitation modes have received broad attention for the accomplishment of robust neuromorphic computing with simple device integration and low energy consumption [156]. Regarding this, optoelectronic synaptic transistors based on CsPbBr_3_ QDs were developed. In early 2019, Wang et al. reported a solution process to blend CsPbBr_3_ QDs with poly(3,3-didodecylquarterthiophene) (PQT-12) as the light-absorbing and charge transport layer in the light simulated synaptic transistors [157]. After integrating QDs with PQT-12, the device performance was substantially improved and synaptic behaviors were observed, including high-pass dynamic filter, pair-pulse facilitation, post-synaptic current, memory, and learning behaviors (Fig. 5b). In 2020, Yang et al. used another organic semiconductor, poly[1,2,5]thiadiazolo[3,4-c]pyridine-4,7-diyl(4,4-dihexadecyl-4Hclopenta[2,1-b:3,4-b0]dithiophene-2,6-diyl)[1,2,5]thiadiazolo[3,4-c]pyridine- 7,4-diyl(4,4-dihexadecyl-4H-cyclopenta[2,1-b:3,4-b0]dithiophene-2,6-diyl) (PCDTPT) to blend with CsPbBr_3_ QDs [158], which enhanced the separation efficiency of photoinduced carriers and the photoresponsivity of the fabricated transistor (Fig. 5c) [158]. Moreover, perovskite QDs can serve as photosensitizers in the thin-film transistor-like synaptic device. In another report, Periyal et al. fabricated a sensitized neuromorphic transistor using amorphous indium gallium zinc oxide (IGZO) semiconductor and CsPbBr_3_ QDs as an active material for brain-inspired computing [159]. This transistor responds to 445 and 525 nm light signals and allows near-linear programming, which is essential to deep learning accelerators in artificial neural networks.

A multifunctional synaptic transistor was reported by Huang’s group in 2021 [160]. The device was built on a ternary organic semiconductor-polymer-inorganic perovskite QDs photosensitive layer. The ternary film consists of C8-BTBT, which has a high crystallinity for the transportation of charge and a strong UV responsivity, CsPbBr_3_ QDs, which have increased UV absorption and charge transfer, and PS polymer, which has a charge-lifetime-enhancement function. The synaptic device demonstrates a low power consumption of ~ 0.11 fJ in one spike under a low operation voltage of − 0.01 V and optically synaptic-like behavior (excitatory postsynaptic current (EPSC), pair-pulse facilitation (PPF), Morse coding) with tunable short-term (STP) and long-term neuroplasticity (LTP). Following this work, Huang's group presented a new way of producing multifunctional and flexible neuromorphic electronics. They applied all solid dielectric materials in organic optoelectronic synaptic transistors, including environmental-friendly ionic conductive cellulose nanopaper (ICCN) and CsPbBr_3_ QDs. The electric modulation came from the electric double layer effect in ICCN, and the photonic modulation was from the heterojunction between CsPbBr_3_ QDs and the organic semiconductor poly[2,5-(2-octyldodecyl)-3,6-diketopyrrolopyrrole-alt-5,5-(2,5-di(thien-2-yl)thieno [3,2-b]thiophene)] (DPP-DTT) [156]. In both electric and photonic modes, synaptic functions can be processed. The transition from short-term potentiation (STP) to long-term potentiation (LTP) happened in either co-modulation of electric and photonic operation or input photonic pulse adjustment. The logic function application in these synaptic transistors was activated by the synergistic modulation of electric and photonic operation. This device also demonstrated outstanding flexibility, as no obvious synaptic performance variation was observed while bent down to a radius of one mm.

In 2022, Ercan et al. investigated the influence of structure and morphology on charge separation at interfaces, transport between semiconducting polymers and QDs, and molecular packing [161]. In addition, they reported composite perovskite-P3HT aggregate systems with preprocessing of solutions for photonic synaptic transistors, which is conducive to small energy-consuming and voltage-driven devices due to high charge dissociation in QDs distributing on nanofibrils. Specifically, they demonstrated a simple path to produce poly(3-hexylthiophene) (P3HT)/perovskite CsPbBr_3_ QD composite nanofibril films (CNFs) with the influence for solution treatments and processing, including ultrasonication, UV light treatment and marginal solvent addition to improve the charge dissociation and photonic synaptic performance. The results revealed that pretreatment with ultrasonication and UV irradiation in the presence of a mixed solvent of chlorobenzene and acetonitrile produces well-packed P3HT polymer chains and restraints the aggregation of CsPbBr_3_ QDs around P3HT nanofibrils. This morphological improvement was shown to be linked to the increased interaction strength that existed between the QDs and the nanofibrils. Such interaction, therefore, led to an increase in charge dissociation in response to photoexcitation, and as a result, the manufactured photonic transistor memory device demonstrated improved performance in terms of photoresponsivity, memory retention discriminability, and *I*_on_/*I*_off_ ratio (~ 10^4^). This QDs/CNF-based synaptic device also showed a super low energy consumption of 0.18 fJ and zero-gate operation, allowing an easy route for developing photonic transistors and synapses for neuromorphic computing.

Apart from directly serving as channel materials, inorganic perovskite QDs can also be blended with conventional nanomaterials and organic semiconductors to modify the transport behaviors. For instance, Pei et al. demonstrated a multifunctional transistor with a stepped floating-gate (FG) of perovskite QDs, where the QD FGs were deposited with the solution-free evaporation method, and the device fabrication was complementary metal–oxide–semiconductor (CMOS) compatible [162]. In order to equip the FG transistor with the photoelectric computing function, they proposed a FET with stepped photoactive FGs and various bandgaps, as demonstrated in Fig. 5d. The transistor showed a bottom-gate top-contact structure by applying CsPbBr_3_ as shallow FG, and CsPbCl_2_Br QDs as the deep FGs. They deposited an Al_2_O_3_ film with accurately controlled thickness, making it tunneling and blocking layers. An amorphous In-Ga-Zn-O (a-IGZO) film was employed as the channel. The transistor exhibited field-effect mobility (μFE) of 8.8 cm^2^ V^−1^ s^−1^, a threshold voltage (*V*_th_) of 0.6 V, an on-to-off current ratio (*I*_on_/*I*_off_) of ∼10^7^, and a subthreshold swing (SS) of 0.44 V dec^−1^. Additionally, this stepped perovskite QD-FGs transistor showed memory behaviors, as shown in Fig. 5d. The stepped FGs transistor exhibited brilliant writing/erasing endurance and memory retention. Noticeably, the device realized logic AND gate computing function by operation with both optical and electrical signals, along with storing output signal simultaneously. Based on these, the convergence of in situ memory and multilevel photoelectric computing was introduced in a single-stepped FGs transistor.

Due to low conductivity, the inorganic perovskite QDs are not suitable to serve as an active layer independently. Several methods have been devised to overcome the issue, including thermal annealing, ligand removal, surface modification, and doping with additives [148]. In 2020, Kim et al. first employed inorganic perovskite QDs in a light-emitting FET without mixing other organic semiconductors or luminophores (Fig. 5e) [163]. This solution-processed CsPbBr_3_ QDs light-emitting FET exhibited a wide recombination zone of 80 µm, electron mobility of 0.12 cm^2^ V^−1^ s^−1^, and an *I*_on_/*I*_off_ ratio of 10^3^ at 517 nm. It is worth mentioning that the Al/ZnO/CsPbX_3_ QDs/poly(9-carbazole)/Au structure demonstrated good air stability. Kim et al. modified CsPbBr_3_ QDs by a sequential methyl acetate solvent soaking and Ostwald ripening process, reducing surface ligands and trap defects. As a result, the mobility of CsPbBr_3_ QDs was dramatically enhanced, and the corresponding FET displayed p-type nature, with mobility of 0.023 cm^2^ V^−1^ s^−1^ and an ON/OFF ratio up to ~ 10^4^ [165]. In addition, Zhou et al. doped Ag^+^ into CsPbBr_3_ QDs at room temperature and applied them in FET [164]. This study suggested that when Ag^+^ partially substitutes Pb^2+^ in CsPbBr_3_ QDs, the Fermi level shifts down to the valence band to induce a p-type character. Thereby, compared with the undoped counterpart, the Ag^+^-doped CsPbBr_3_ QDs-based FET showed an increase of three orders of magnitude in hole mobility (Fig. 5f), which was attributed to suppressed ion transportation and phonon screening. Furthermore, this group applied the Ag^+^ and Bi^+^ co-doped CsPbBr_3_ QDs film in FETs to study the electronic-ionic transport [143]. They concluded that the electronic transport is decoupled from the ionic transport in QD films, resulting in a unipolar transport property in a p-type mode with a well-defined linear saturation regime when the temperature is below 240 K. Moreover, doping Bi^+^ lifts the Fermi level (*E*_F_) of CsPbBr_3_ QDs and switches the device to a normally off (enhancement) p-channel mode. On the contrary, doping Ag^+^ moves down E_F_ and switches the device to a normally on (depletion) state.

Due to superior absorption properties and high conversion efficiency, inorganic perovskite QDs can also be used as promising dopants to promote the characteristic of channel medium and device performance. For example, Lee et al. adopted CsPbX_3_ QDs as the dopant in MSe_2_ (M = Mo and W) FET [166]. They hybridized the green-light-emitting CsPbBr_2_I QDs and blue-light-emitting CsPb(Cl/Br)_3_ QDs with MoSe_2_ and WSe_2_ layers in 2D MSe_2_-based FET to improve the photoresponse characteristics. The WSe_2_, MoSe_2_, WSe_2_/CsPbBr_2_I_1_-QDs, and MoSe_2_/CsPb(Cl/Br)_3_-QDs FET devices were measured, and it was found that the p-channel current decreased and n-channel current increased upon light irradiation after mixing with inorganic perovskite QDs. Moreover, because the trapped hole induces the photogate effect, the threshold voltage negatively shifts. Other than FET, a novel adaptive device based on a mixed-dimensional van der Waals heterostructure (MvdWHs) of gate-modulated 0D-CsPbBr_3_-QDs/2D-MoS_2_ transistor was fabricated by Xie et al. [167]. Historically, the investigation of visual adaptive devices centered mostly on the construction of circuits using silicon-based transistors as logic devices, which have played a crucial role in artificial adaptive fields. However, these strategies may be ineffective for large-scale device integration owing to their enormous space requirements and high energy consumption. Therefore, MvdWHs have been presented as a potential device design because of their high carrier mobility, large surface-to-volume ratios, and inherent optoelectronic features. Particularly, it has been established that 0D–2D MvdWHs offer a number of benefits, including ultrathin material thickness, exceptional photoabsorption capabilities, and less restricted lattice matching difficulties. The CsPbBr_3_-MoS_2_ MvdWHs-based adaptive phototransistor shows remarkable optoelectronic properties due to the intense light absorption capabilities of CsPbBr_3_ QDs on the top and the good carrier transport abilities of 2D MoS_2_ at the bottom. The comparisons among various perovskite QDs transistors and their performance published during the past few years are shown in Table 2.

**Table 2:** Comparison of performance parameters for perovskite QD-based transistors

Year	Composition	Other material in the channel	*μ*_*n*_ (cm^2^ V^−1^ S^−1^)	*μ*_*h*_ (cm^2^ V^−1^ S^−1^)	*I*_on_/*I*_off_	Refs.
2017	CsPbI_3_	Poly[9,9-bis-(2-ethylhexyl)-9H- fluorene-2,7-diyl] (PFO)	–	2.4 × 10^−1^	–	[153]
2017	CsPbBr_3_	Dinaphtho[2,3-b:2', 3' -f]thieno[3,2-b] thiophen (DNTT)	–	–	8.1 × 10^4^	[154]
2019	CsPbBr_3_	Ag doping	–	8 × 10^−4^	–	[164]
2020	CsPbBr_3_	Ag & Bi doping	–	~ 10^−3^	~ 10^4^	[143]
2020	CsPbBr_3_	Indium gallium zinc oxide (IGZO)	5.07	–	1 × 10^6^	[159]
2020	CsPbBr_3_	–	1.2 × 10^−1^	–	1 × 10^3^	[163]
2020	CsPbBr_3_	–		2.3 × 10^−2^	1 × 10^4^	[165]
2020	CsPb(Cl/Br)_3_	MSe_2_ (M = Mo and W)	6.1 × 10^−2^	–	–	[166]
2021	CsPbBr_3_/ CsPbCl_2_Br	In-Ga-Zn–O	–	–	~ 10^7^	[162]
2022	CsPbBr_3_	Poly(3-hexylthiophene) (P3HT)	–	2.9 × 10^−4^	~ 10^4^	[161]

### Inorganic Perovskite QDs Based Memories

The memory speed has increased by around 10% each year, whereas the processor speed has increased by approximately 55% per year on average during the previous two decades, resulting in a seriously unbalanced lagging of memory development behind the processor's computing speed. This limitation of processor speed, due to the constraint of memory performance, is called “Memory Wall” [158, 168]. Accordingly, new types of device architectures have been designed and built [169, 170], including resistive switching random access memory (RRAM) [171], phase-change memory (PCM) [172], nonvolatile floating-gate transistor memory (NVFGM) [173], and ferroelectric random access memory (FRAM) [174]. Among them, RRAM shows excellent potential for neuromorphic systems, logic operation, and data storage. It has advantages of excellent scalability, easy fabrication, long data retention, simple metal–insulator-metal (MIM) structure, and nanosecond speed, thus providing lots of potential for advancing memories [175]. In general, RRAM has a conductor/insulator/conductor sandwich structure [176]. The active layer such as inorganic perovskite QDs serves as the media for ion transport and storage, and the RRAM resistance can be reversibly switched between a high resistance state and low resistance state (corresponding to 0 and 1 in the logic perspective) by applying external voltages due to the formation/rupture of conducting filaments or altering of the electrode/insulator interface. The threshold voltage triggering the switching from high to low resistance state is the set voltage, and vice versa is the reset voltage.

Metal halide perovskites are suitable for RRAM applications because they contain abundant ionic point defects like interstitials, substitutions, and vacancies with low formation energies [177–179]. The migration of these ionic defects is a double-edged sword for general perovskite-based electronics, causing hysteresis and poor stability in solar cells [180], while leading to resistance switching in memory devices [181–184]. Since metal halide perovskites possess a sensitive response to light, light excitation can be another parameter in addition to the electric field to effectively control the resistance states [185–187]. The mechanisms responsible for resistive switching effects in metal halide perovskites can be divided into several categories related to phenomena such as formation/rupture of filaments from electrodes, metal cation-induced filaments, ions migration, and trap-controlled space-charge limited current (SCLC).

**Fig. 6:**
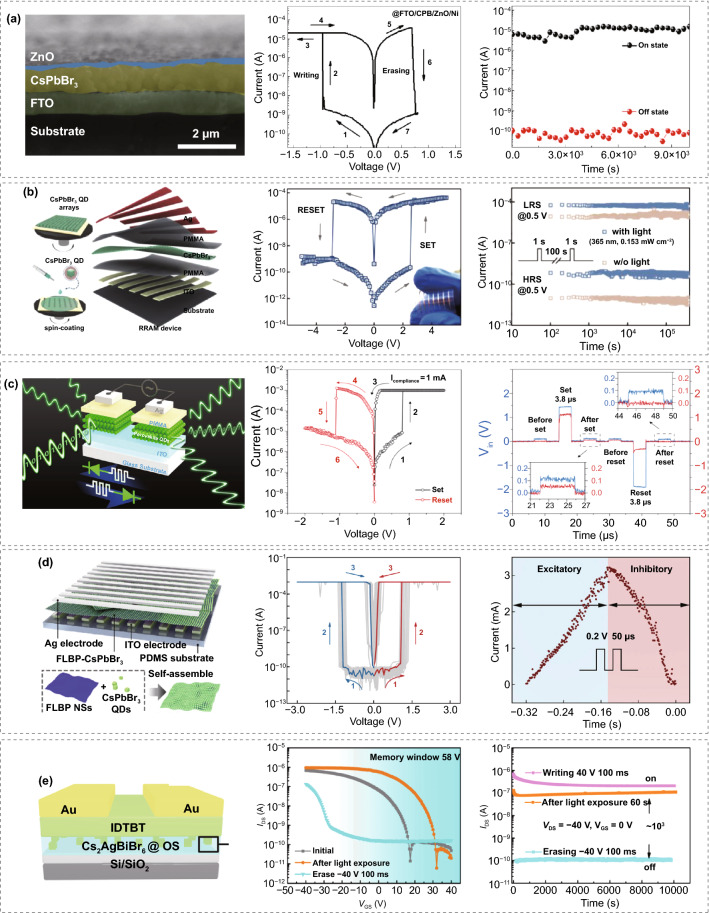
**a** Cross-sectional SEM image of the Ni/ZnO/CsPbBr_3_ QDs/FTO memristor, typical *I–V* curves, and retention measurement. Adapted with permission from Ref. [188]. Copyright 2017, Springer. **b** Schematic diagram of CsPbBr_3_ QD-based logic OR device, its photonic RRAM *I–V* plot and retention performance. Adapted with permission from Ref. [185]. Copyright 2018, Wiley–VCH. **c** Schematic diagram of CsPbBr_3_ QD-based LEM device with dual functionalities, along with the device *I–V* characteristic and transient responses of the RRAM during the set and reset processes. Adapted with permission from Ref. [191]. Copyright 2020, Nature Portfolio. **d** Schematic diagram of the FLBP–CsPbBr_3_ TSM device structure, its *I-V* characteristics, and its excitatory and inhibitory response. Adapted with permission from Ref. [194]. Copyright 2021, Nature Portfolio. **e** Schematic diagram of the Cs_2_AgBiBr_6_ QDs@OS-based photomemory structure, transfer characteristics and retention results. Adapted with permission from Ref. [195]. Copyright 2020, American Chemistry Society

In a pioneering work, Wu et al. demonstrated a ZnO/CsPbBr_3_ QDs-based memristor (Fig. 6a), featuring a large ON/OFF ratio (> 10^5^) and a low working voltage (< 1 V) [188]. To improve the switching stability, An et al. made memory devices via blending CsPbCl_3_ with PMMA, a widely used matrix material in memristive devices due to its dielectric properties and environmental resistance to enhance the device stability [189]. The PMMA can catch electrons to generate a high internal field, while the CsPbCl_3_ QDs serve as traps in the PMMA matrix, forming local conducting paths.

Among efforts on photonic RRAMs, Han's group fabricated CsPbBr_3_ QDs as the active layer in an ITO/PMMA/CsPbBr_3_ QDs/PMMA/Ag structure (Fig. 6b) [185]. Here, the formation and annihilation of conductive filaments lead to resistive switching, which is attributed to the migration of Ag ions and Br defects in QDs driven by the external electric field and light irradiation. The RRAM device in this study also functioned as an "OR" logic gate, integrating electric field and light as input signals. The group also found that the set/reset voltages could be effectively reduced by increasing the light intensity. An RRAM-gate FET analogous flash memory was demonstrated after the CsPbBr_3_ QDs-based RRAM was coupled with a p-channel transistor. Following this work, Chen et al. demonstrated a simpler sandwich structure of all inorganic perovskite QDs-based RRAM (Au/CsPbBr_3_ QDs/ITO) by solution-processed method [190]. The resistive switching effect of this device comes from the connection and rupture of conducting filaments formed by Br vacancies under the electric field. This memory device exhibited light-assisted multilevel storage and long data retention of 1000 s with high reproducibility. The ON/OFF ratio reached ~ 10^7^, with a low reading voltage of − 0.3 V.

Recently, Yen et al. demonstrated an interesting all-inorganic perovskite QD light-emitting memory (LEM) [191]. In their work, a single Ag/PMMA/CsPbBr_3_ QDs/ITO device can switch between RRAM and light-emitting electrochemical cell on a sub-millisecond scale by modulating its bias polarity (Fig. 6c). The RRAM function provided an electrical reading of the encoded data, while the light-emitting memory function can be used for a parallel, non-contact optical reading at a speed of 5 kHz. During the operation of a light-emitting electrochemical cell (LEC), the cations and anions are dissociated from CsPbBr_3_ QDs and drift to Ag cathode and ITO anode, respectively, resulting in band bending of the CsPbBr_3_ QDs at the interfaces and the formation of a p-i-n diode, which facilitates the photon emission through the radiative recombination of electron–hole pairs. The RRAM showed retention of over 10^5^ s and an On/Off ratio of around 10^2^.

In addition to RRAM, the photonic synapses have aroused lots of interest. Han's group used QDs to build a photonic synapse device with a structure of Au/pentacene/PMMA/CsPbBr_3_ QDs/SiO_2_/Si [192]. In this work, the photonic potentiation was induced by light, and the device showed a depression behavior under electrical bias. The separation of excitons at the interfaces of pentacene and CsPbBr_3_ QDs leads to light-induced charge trapping and releases in the photonic flash memory. Moreover, long-term plasticity, short-term plasticity, and spike-rate-dependent plasticity were modulated by the optical source to emulate the biological synaptic functions. Two years later, Huang's group successfully fabricated a photonic synaptic device based on the inorganic perovskite QDs/organic semiconductor composite, which realized fundamental synaptic behaviors, including the transition of short-term memory to long-term memory, learning experience, pair-pulse facilitation, and excitatory postsynaptic current [193]. The photonic synaptic was constructed with a simple configuration, blending CsPbBr_3_ QDs with organic semiconductor (poly[2,5-(2-octyldodecyl)- 3,6-diketopyrrolopyrrole-alt-5,5-(2,5-di(thien-2-yl)thieno [3,2-b] thiophene)], DPPDTT) in solution and then spin-coating the hybrid film as the conduction layer. The photoinduced electrons can be trapped in CsPbBr_3_ QDs, while holes are transferred to the DPPDTT, subsequently forming a built-in electric field and further turning on the photogating effect. The CsPbBr_3_ QDs in the device provided an enhancement of photosensitivity, while the organic semiconductor increased the output signal strength. Hence, this device could work under a low operation voltage, presenting synaptic performances at – 0.2 V and showing obvious synaptic response under an ultralow operation voltage of – 0.0005 V. Furthermore, both “AND” and “OR” logic functions were demonstrated with the regulation of the synaptic input parameters.

In 2022, Kovalenko's group reported a reconfigurable CsPbBr_3_ QDs memristor with both volatile diffusive and multistate nonvolatile drift kinetics, exhibiting facile switching between the two modes and remaining pertaining good performance for neuromorphic computing [142]. The reconfigurable memristive computing substrate enables the active control of drifts and ionic diffusive dynamics. Hence, they applied silver as an active electrode and comprised organic ligands capping CsPbBr_3_ QDs as an active switching matrix. The device was structured with indium tin oxide (ITO), poly (3,4-ethylenedioxythiophene) poly-styrene sulfonate (PEDOT:PSS), poly(*N*,*N*’-bis-4-butylphenyl-*N*,*N*’- bisphenyl)benzidine (polyTPD), CsPbBr_3_ QDs and Ag. In this structure, the migration of Br^−^ and Ag^+^ with low activation energy made it simple for conductive filaments to form, and the soft lattice of CsPbBr_3_ QDs sped up the diffusion of mobile ions. Besides, organic capping ligands have the capability of assisting in the regulation of electrochemical processes, which results in excellent reconfigurability and exceptional durability. In addition, the selection of capping ligands determined the endurance performances in both volatile and nonvolatile modes. Comparative analysis was performed on two different types of capping ligands, didodecyldimethylammonium bromide (DDAB) and oleylguanidinium bromide (OGB). CsPbBr_3_ QDs capped with DDAB ligands displayed poor switching behavior in both volatile (10 cycles) and nonvolatile (50 cycles) modes. In contrast, CsPbBr_3_ QDs with OGB ligands exhibited the highest endurance performances among the reports to date in both volatile and nonvolatile switching modes, which can achieve 2 × 10^6^ cycles and 5.6 × 10^3^ cycles, respectively.

Moreover, a biomimetic compound eye based on an artificial lobula giant movement detector (LGMD) visual neuron is implemented using a light-mediated threshold switching memristor (TSM), as reported by Han's group [194]. The TSM array is composed of a single device structure with Ag/few-layer black phosphorous nanosheets-CsPbBr_3_ QDs heterostructure (FLBP-CsPbBr_3_)/indium tin oxide (ITO) (Fig. 6d). LGMD, which is sensitive to movement with a wide-field visual neuron in the third visual neuropile of lobula, can react to looming objects and trigger escape behavior rapidly. Han’s group also developed a new LGMD-inspired artificial neuron based on a single two-terminal volatile FLBP/CsPbBr_3_ TSM [194]. Such a device showed an extremely large ON/OFF ratio of over 10^7^ and the typical *I–V* characteristics as shown in Fig. 6d. The excitatory and inhibitory responses to the light flow field were triggered by the formation and deconstruction of Ag-conductive filaments (CFs), resembling the escape response of LGMD neurons. Besides, the biomimetic compound eye displayed 180° × 180° wide field-of-view (FoV) detection capability and nonmonotonic collision avoidance response toward looming stimuli.

Considering the environmental-friendly issue, researchers attempted substituting lead components in inorganic perovskite QDs for memory devices. Currently, since the memory devices based on inorganic perovskite QDs are still in the early stages of development, the research on lead-free perovskite QDs remains limited. Lin et al. were the first to pioneer lead-free perovskite QDs-based memory [195]. In their work, they wrapped the Cs_2_AgBiBr_6_ QDs within the oligomeric silica (OS) matrix and polyvinylpyrrolidone (PVP), respectively, as a charge tunneling layer for floating gate transistor photomemory (FGTPM). Compared with the traditional floating gate layer structured Cs_2_AgBiBr_6_@PVP, Cs_2_AgBiBr_6_@OS showed a significant improvement in retention capacity with a larger memory window of 58 V (Fig. 6e). The OS-wrapped Cs_2_AgBiBr_6_ QDs composite layer was used as the hybrid floating gate and charge tunneling layer, in which Cs_2_AgBiBr_6_ QDs were applied as photosensitizers. In addition, the thickness of the OS layer can be controlled in spin-coating to regulate the photomemory retention capacity. A comparison between various perovskite QDs memories and their performance over the past years is shown in Table 3.

**Table 3:** Comparison of structure and performance for perovskite QD-based memories

Year	Composition	QD treatment	Electrode	Other material in the channel	Set/reset voltage (V)	Power consumption (W)	On/off ratio	Retention (s)	Mechanism	Refs.
2017	CsPbBr_3_	–	Ni/FTO	ZnO	+ 0.95/ + 0.71	< 10^−6^	> 10^5^	10^4^	Interface-type	[188]
2018	CsPbCl_3_	Mixing with PMMA	Al/ITO	PMMA	− 0.3/2.6	–	2 × 10^4^	1 × 10^4^	Trap-controlled SCLC	[189]
2018	CsPbBr_3_	Annealing at 140 °C for 40 min	Ag/ITO	PMMA	+ 2.3/ − 2.7 (w/o light) 1.1/ − 1.7 (with light)	< 10^−4^	10^5^	4 × 10^5^	Ag and V_Br_ CFs	[185]
2018	CsPbBr_3_	Annealing at 140 °C for 40 min	Au/Si/ SiO_2_	Pentacene/PMMA	–	1.4 × 10^−9^	1.86 × 10^5^	~ 10^7^	Charge trapping/detrapping	[192]
2019	CsPbBr_3_	–	Au/ITO	–	− 3.9/ − 0.4	–	10^7^	10^3^	Trap‐filled SCLC	[190]
2020	CsPbBr_3_	Mixing with DPPDTT/Annealing at 100 °C for 30 min	Au (Si/SiO_2_ Gate)	DPPDTT	− 0.0005 V	–	–	–	Charge trapping/detrapping	[193]
2021	CsPbBr_3_	–	Au/ITO	PMMA	+ 0.7/ − 1.1	–	~ 10^2^	10^5^	Ag and V_Br_ CFs	[191]
2021	CsPbBr_3_	Annealing at 100 °C for 30 min	Au/ITO	Few-layer black phosphorous nanmixosheets (NSs)	+ 3/ − 3	4.1 × 10^−7^	> 10^7^	–	Ag CFs	[194]
2022	CsPbBr_3_	Capped with didodecyldimethylammonium bromide (DDAB) and oleylguanidinium bromide (OGB) ligands	Au/ITO	PEDOT:PSS/ polyTPD	+ 1/ − 0.8	–	≥ 10^3^	10^5^	Ag and V_Br_ CFs	[142]
2020	Cs_2_AgBiBr_6_	Mixing with tetramethoxysilane (TOMS)/ Annealing at 60 °C for 20 min	Au/Si/SiO_2_	Oligomeric silica (OS)/ polyvinylpyrrolidone (PVP)	–	–	–	10^3^	Floating gate	[195]

## Conclusion and Outlook

In this review, we summarized the physical properties and synthesis methods of all-inorganic perovskite QDs and systematically discussed the recent research works on their electronic applications. Inorganic perovskite QDs have notable advantages, such as defect tolerance, narrow FWHW, and high synthesis feasibility compared to traditional QDs (e.g., CdS, CdSe, PbSe, and SnS). With low-cost solution processing fabrication, this material family offers a new possibility for applications in large-area flexible and stretchable electronic devices. Thanks to their higher melting point, stronger photostability, and thermostability than organic and hybrid counterparts, inorganic perovskite QDs are becoming rising stars in the new era of nanoelectronics.

However, there are some challenges concerning the applications of inorganic perovskite QDs-based electronics. First, although inorganic perovskite QDs were predicted to possess a long diffusion length larger than 9.2 μm and high carrier mobility around 4500 cm^2^ V^−1^ s^−1^ in theoretical simulation, experimental verification has been lagged behind and there still exists a huge gap between theory and reality [85]. Complicated surface chemistry and poor electronic coupling are the main limitations of the performance of QD-based electronic devices [196]. For example, ligands are a double-edged sword for inorganic perovskite QDs. They can ensure stability but also produce defects [197]. Further, the long and insulating ligands also trap charges in the inorganic perovskite QDs and expand the interparticle distance, leading to poor electronic coupling. Therefore, ligand engineering is one research focus for advancing inorganic perovskite QD electronics, including strategies of ligands exchange [198], ligands passivation [199], ligand-mediated ion exchange [200].

Second, the long-term stability of inorganic perovskite QDs in a harsh environment remains an urgent issue to be solved. In addition, the toxicity of lead in inorganic perovskite QDs is a pressing issue that requires significant attention. Recently, research has been conducted on exploring new alternative lead-free compounds. For example, Cs_3_Bi_2_X_9_ [201], CsSnX_3_ [202], Cs_3_Sb_2_Br_9_ [203], Cs_2_AgBiX_6_ [204], and Cs_2_AgInCl_6_ [205] QDs have been successfully synthesized and shown great potential to resolve the toxicity issue [206]. However, their optical and electronic properties remain inferior to lead-based inorganic perovskite QDs, thus restricting their application in high-performance electronics.

The quality of inorganic perovskite QD layers, especially their uniformity, should be carefully controlled since the memory performance depends on the thickness. The memory device built on thicker inorganic perovskite QD films requires a larger voltage to be switched on, but the device made with thinner films often displays an unstable resistive switching performance. Thus, special consideration should be given to depositing QDs films with appropriate thickness and high uniformity to improve the performance and reliability of electronics.

Regarding the industry-scale processing of inorganic perovskite QDs, solvent toxicity could be a challenge. The common solvents for dispersing inorganic perovskite QDs are hexane and toluene, which are harmful to humans and toxic to the environment. Recently, a techno-economic model suggested that solvent recycling can be cost-efficient and eco-friendly for industrial scale-up [207]. Besides, owing to the low boiling points of hexane and toluene, they can evaporate extremely fast during the film manufacturing process, causing the "coffee ring" effect and affecting the homogeneity of as-fabricated films. Consequently, research into alternate ecologically acceptable solvents that have the appropriate boiling temperatures and the compatibility with inorganic perovskite QDs should be pursued.

Moreover, to achieve the commercialization of QD electronics, it is imperative to produce large-area films; however, the conventional spin-coating approach is unable to satisfy this need. As a result, other methods of producing inorganic perovskite QD films for electronics, like ink-jet printing and spray coating, may be used [208]. More research efforts are warranted to develop these scalable synthesis methods in the context of QD electronics. Specifically, finding appropriate solvents is one of the significant challenges for ink-jet printing of inorganic perovskite QDs [209]. Typical solvents used for Cd-based QDs, such as ether or ketones, will cause precipitation of inorganic perovskite QDs. Instead, newly designed novel inks containing n-tridecane, n-nonane, or naphthene for inkjet-printing inorganic perovskite QDs were reported to be able to generate a high-quality thin film with fewer surface defects.

Lastly, flexible electronics based on inorganic perovskite QDs are a prospective research topic that should be pursued. Inorganic perovskite QDs have dimensions on the nanoscale, making them more adaptable to flexible applications than their bulk counterparts. Recently, highly flexible perovskite QD solar cells with excellent bending durability were reported [210], while a wide range of flexible electronics based on these emerging QDs clearly warrants future research efforts. In addition to the adaption of inorganic perovskite QDs for the substrate, the barrier materials for encapsulation and their mechanical durability, surface hydrophobicity, water vapor, and oxygen transmission rate are imperative for preventing perovskite from degrading [211]. Currently, the promising materials are flexible glass [212], polymer nanocomposites [213], and thin-film barriers that are comprised of inorganic single-layers such as AlO_x_, SiO_x_, TiO_2_, SnO_x_, and SiN [214, 215]. However, these materials have not reached commercial standards, and further developments are needed for large-scale applications.

In the past decade, perovskite-based electronics have rapidly advanced, yet research works on inorganic perovskite QD-focused are lagging behind. As a result, there is still a significant amount of uncharted territory to be explored regarding inorganic perovskite QDs-based FETs, artificial synapses, and RS memory devices. Considering the blooming research activities on bulk perovskites and the unique advantages of perovskite QDs, we believe that inorganic perovskite QDs will ignite the next wave of activities on advancing high-performance nanoelectronics and disruptive technologies.
